# Interleukin 10 level in the peritoneal cavity is a prognostic marker for peritoneal recurrence of T4 colorectal cancer

**DOI:** 10.1038/s41598-021-88653-2

**Published:** 2021-04-28

**Authors:** Seung-Yong Jeong, Byeong Geon Jeon, Ji-Eun Kim, Rumi Shin, Hye Seong Ahn, Heejin Jin, Seung Chul Heo

**Affiliations:** 1 Department of Surgery, Seoul National University College of Medicine, Seoul, Republic of Korea; 2 Department of Surgery, Daejin Medical Center Bundang Jesaeng General Hospital, Sungnam-Si, Republic of Korea; 3 Department of Surgery, Seoul National University-Seoul Metropolitan Government (SNU-SMG) Boramae Medical Center, Seoul, Republic of Korea; 4 Medical Research Collaborating Center, Seoul National University-Seoul Metropolitan Government (SNU-SMG) Boramae Medical Center, Seoul, Republic of Korea

**Keywords:** Cancer, Immunology

## Abstract

Peritoneal recurrence (PR) is a major relapse pattern of colorectal cancer (CRC). We investigated whether peritoneal immune cytokines can predict PR. Cytokine concentrations of peritoneal fluid from CRC patients were measured. Patients were grouped according to peritoneal cancer burden (PCB): no tumor cells (≤ pT3), microscopic tumor cells (pT4), or gross tumors (M1c). Cytokine concentrations were compared among the three groups and the associations of those in pT4 patients with and without postoperative PR were assessed. Of the ten cytokines assayed, IL6, IL10, and TGFB1 increased with progression of PCB. Among these, IL10 was a marker of PR in pT4 (N = 61) patients based on ROC curve (*p* = 0.004). The IL10 cut-off value (14 pg/mL) divided patients into groups with a low (7%, 2 of 29 patients) or high (45%, 16 of 32 patients) 5-year PR (*p* < 0.001). Multivariable analysis identified high IL10 levels as the independent risk factor for PR. Separation of patients into training and test sets to evaluate the performance of IL10 cut-off model validated this cytokine as a risk factor for PR. Peritoneal IL10 is a prognostic marker of PR in pT4 CRC. Further research is necessary to identify immune response of intraperitoneal CRC growth.

## Introduction

Peritoneal carcinomatosis (PC), which is a major cause of death following hematogenous metastasis in patients with colorectal cancer (CRC)^1^, develops in 10–35% of curatively resected cases^2,3^. In most cases, PC is intractable, as systemic chemotherapy has little effect, and cytoreductive surgery with hyperthermic intraperitoneal chemotherapy is effective in very few patients^4^. Numerous studies have attempted to predict postoperative peritoneal recurrence (PR) to facilitate early detection and the administration of relevant adjuvant therapy to high-risk patients. Most of these studies aimed to detect and quantify free peritoneal cancer cells using cytological or molecular methods^5,6^; however, the effectiveness of using free peritoneal cancer cells to predict PR is unclear^7^. Moreover, peritoneal cancer cells are usually to predict systemic recurrence rather than PR specifically.

The advent of cancer immunotherapy based on the blockade of the PD-1/PD-L1 interaction opened up a new era of cancer treatment^8^. The efficacy of anti-PD-1 agents demonstrated that interactions with the immune system are vital for cancer growth. CRC was one of the earliest cancers for which prognosis was shown to be influenced by the immune system^9^, and the ‘Immunoscore’ was validated in an international study^10^; however, anti-PD-1 agents are ineffective for most CRC cases^11^. Therefore, according to the cancer immunoediting theory, mechanisms of immune suppression other than the PD-1/PD-L1 axis must exist in CRC^12^.

The peritoneal cavity is an immunologically active organ in which diverse immune cells and immune proteins interact with cancer cells that invade the peritoneal cavity. Therefore, we hypothesized that the immune system influences PR in CRC and that immune factors can indicate peritoneal tumor growth and act as predictors of PR after CRC resection.

The identification and measurement of immune cells and immune proteins in peritoneal fluid (ascites) can characterize the immune status in the peritoneal cavity. In addition, we can specify immune characteristics according to the stages of peritoneal cancer burden based on pathological results as follows: no tumor cells (pT3 or lower; ≤ pT3), microscopic tumor cells (pT4), and gross tumors (M1c). Moreover, because the peritoneal cavity is substantially sterile, unless bowel perforation or intraperitoneal abscess occurs, this model is free from interference by immune responses to commensal bacteria, which is impossible in primary tumors.

Here, we designed a peritoneal tumor growth model and performed a pilot study to test our hypothesis and to validate this model by evaluating immune cytokines in ascites collected from patients with CRC undergoing surgery.

## Results

**Figure 1:**
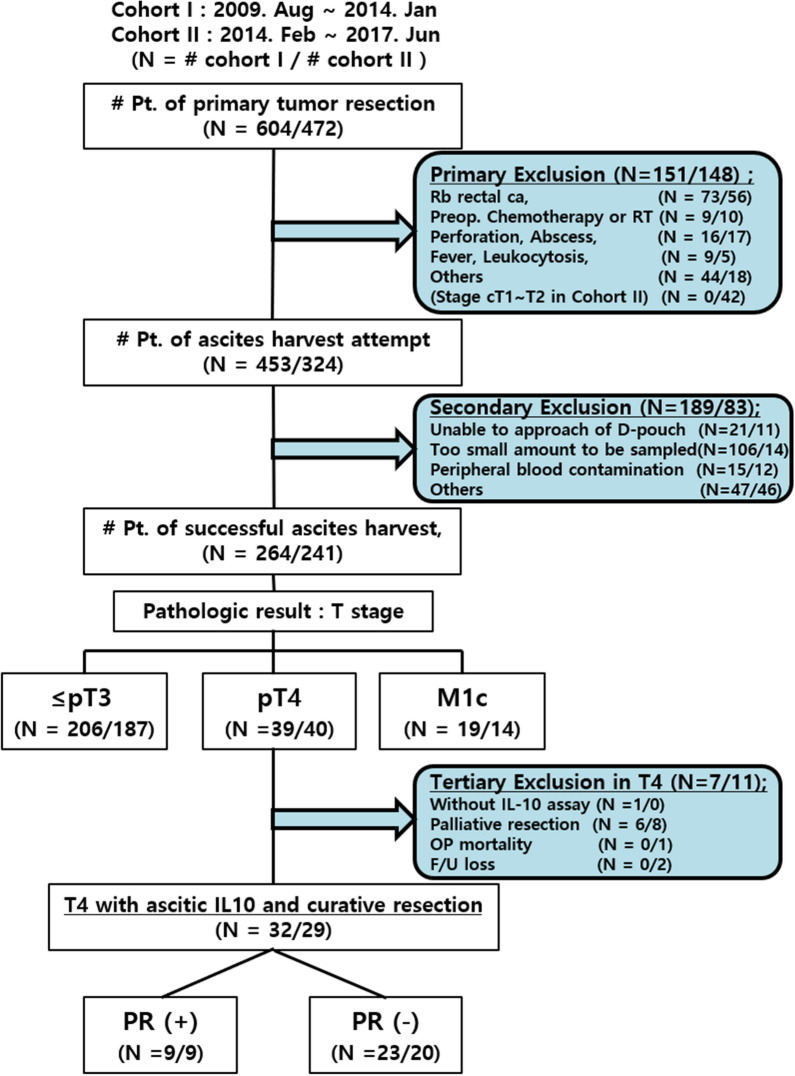
Patient enrolment and exclusion criteria for ascites sampling and assessment of peritoneal recurrence. To investigate the relationship between peritoneal tumor burden and peritoneal immune characteristics, ascites was collected from patients undergoing surgery for colorectal adenocarcinoma since August 1, 2009. Patients with the following peritoneal conditions that could have influenced the results were excluded: patients with extraperitoneal rectal cancer (Rb rectal cancer) determined by pelvic MRI and operative findings (the lower margin located below the anterior peritoneal reflection); those who had undergone preoperative chemotherapy or radiotherapy; and those with intestinal perforation, abscess, leukocytosis, or fever (over 37.3 °C, two or more consecutive times at 4-h intervals within 24 h). Patients who did not agree to take part in the study were also excluded (primary exclusion). By January 31, 2014 (cohort I), we collected ascites from a sufficient number of patients to identify trends in cytokine distribution in three groups of patients: ≤ pT3, pT4, and M1c. Therefore, we did not attempt ascites sampling from patients with probable T1 or T2 (clinical stage T1 or T2) stage tumors from February 1, 2014 (cohort II), as it was unnecessary to have ascites samples from so many patients with ≤ pT3, while patients with clinical T3 tumors were not excluded because it was possible that those tumors would be classified as T4 on pathological examination. Ascites harvest was attempted in all other patients who were not subject to primary exclusion; however, there were some failures of ascites sampling due to adhesions, insufficient ascites, or blood contamination of ascites, and these patients were also excluded (secondary exclusion). Furthermore, patients undergoing palliative resection (R1 or R2 resection), those with operative mortality (who died within 30 postoperative days), and those for whom no postoperative surveillance imaging was conducted (follow-up loss) were not surveyed for peritoneal recurrence (tertiary exclusion in the pT4 group).

Ascites were harvested and cryopreserved at the time of operation from patients meeting the inclusion criteria since August 1, 2009 (cohort I, Fig. 1). The patients were grouped according to peritoneal cancer burden based on the pathological reports. Because there was a large number of patients with ≤ pT3, we did not collect ascites from patients in clinical stage T1 or T2 since February 1, 2014 (cohort II).

### IL6, IL10, and TGFB1 in ascites increase with increasing peritoneal cancer burden

All pT4 (39 patients) and M1c (19 surgical and 7 nonsurgical patients) patients and only the initial 78 (double the number of patients in the pT4 group) ≤ pT3 patients from cohort I were included in the analysis of ten cytokines. Therefore, samples from a total of 143 patients (102 male and 41 female) were eligible for analysis of immune cytokines and peritoneal tumor burden. The mean patient age was 66.5 ± 11.9 years (Table 1).

**Table 1:** Clinical characteristics and peritoneal cytokine concentrations^§^ according to peritoneal cancer burdens. (143 patients).

	pT3 or less (n = 78)	pT4 (n = 39)	M1c (n = 26)	*P*
Age, years (mean ± SD)	65.7 ± 12.3	68.8 ± 10.7	65.4 ± 12.4	0.359
**Gender**				0.243
Male	60 (76.9%)	26 (66.7%)	16 (61.5%)	
Female	18 (23.1%)	13 (33.3%)	10 (38.5%)	
**Tumor location**				0.340
A-colon	19 (24.4%)	13 (33.3%)	9 (34.6%)	
T-colon	6 (7.7%)	5 (12.8%)	0 (0.0%)	
D-colon	5 (6.4%)	3 (7.7%)	2 (7.7%)	
S-colon	17 (21.8%)	8 (20.5%)	10 (38.5%)	
Upper rectum	31 (39.7%)	10 (25.6%)	5 (19.2%)	
**TNM stage**				< 0.001
I	12 (15.4%)	0 (0.0%)	0 (0.0%)	
II	33 (42.3%)	15 (38.5%)	0 (0.0%)	
III	28 (35.9%)	15 (38.5%)	0 (0.0%)	
IV	5 (6.4%)	9 (23.1%)	26 (100)	
IL2 (pg/mL)	4.4 (3.9, 5.2)	4.6 (4.0, 5.1)	5.2 (4.5, 6.0)	0.011
IL4 (pg/mL)*^,‡^	1.1 (0.8, 1.9)	1.9 (0.7, 2.3)	2.7 (2.4, 3.8)	< 0.001
IL5 (pg/mL)	4.4 (4.1, 5.3)	3.8 (3.4, 6.4)	4.8 (4.1, 9.5)	0.017
IL6 (pg/mL)*^,‡^	123.2 (35.7, 418.0)	260.9 (51.5, 1159.1)	2,149.0 (731.5, 6,225.0)	< 0.001
IL10 (pg/mL)*^,‡^	13.8 (9.1, 19.2)	18.4 (10.7, 34.9)	94.1 (30.9, 195.3)	< 0.001
IL12 (pg/mL)*^,†^	2.2 (2.1, 2.7)	2.9 (2.8, 3.1)	3.0 (2.9, 3.1)	< 0.001
IL17 (pg/mL)	4.7 (4.1, 6.1)	4.4 (4.0, 7.1)	6.1 (4.5, 6.8)	0.051
IFNG (pg/mL)	4.9 (4.0, 7.1)	5.8 (4.5, 8.6)	6.2 (5.2, 7.3)	0.041
TNF (pg/mL)	2.3 (2.2, 2.6)	2.2 (2.1, 2.8)	2.4 (2.1, 3.1)	0.245
TGFB1 (pg/mL)*	172.2 (95.3, 259.9)	231.8 (142.1, 345.8)	287.5 (201.0, 646.3)	0.002

*A* ascending, *T* transverse, *D* descending, *S* sigmoid, *IL* interleukin, *IFNG* interferon gamma, *TNF* tumor necrosis factor, *TGFB1* transforming growth factor beta 1.

*p < 0.005, Kruskal–Wallis test for pT3 or less vs. pT4 vs. M1c.

^†^p < 0.0025, Bonferroni adjusted p-value by Mann–Whitney U test for pT3 or less vs. pT4 (reference), two-tailed.

^‡^p < 0.0025, Bonferroni adjusted p-value by Mann–Whitney U test for pT4 (reference) vs. M1c, two-tailed.

^§^Median value (first quartile, third quartile).

**Figure 2:**
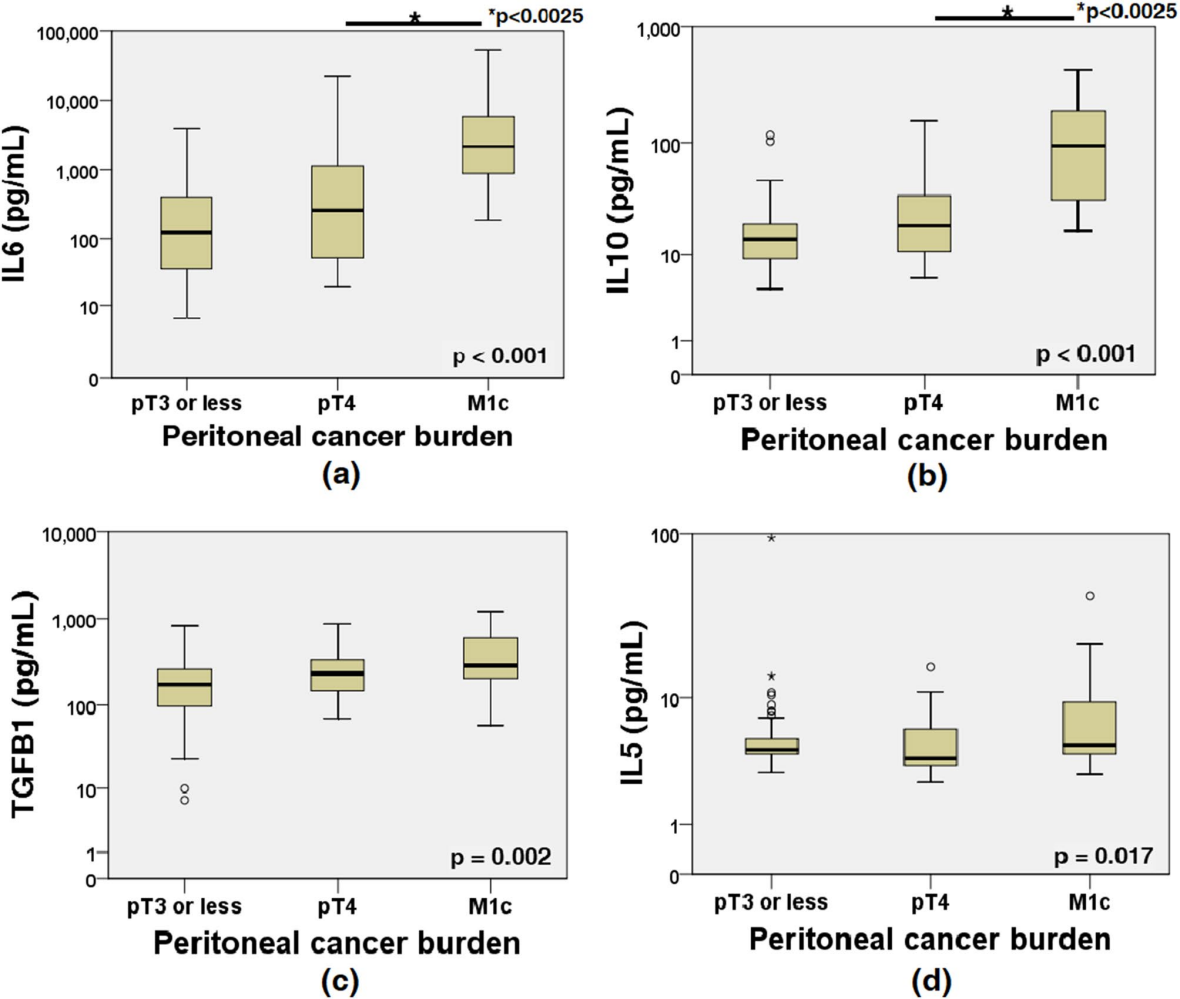
Cytokine concentrations in ascitic fluid according to peritoneal cancer burden. (**a**) Interleukin (IL)6 increased according to peritoneal cancer burden (p < 0.001 in K–W; p = 0.021 for ≤ pT3 vs pT4 and p < 0.001 for pT4 vs M1c in M–U). (**b**) IL10 increased according to peritoneal cancer burden (p < 0.001 in K–W; p = 0.030 for ≤ pT3 vs pT4 and p < 0.001 for pT4 vs M1c in M–U). (**c**) TGFB1 increased according to peritoneal cancer burden (p = 0.002 in K–W; p = 0.023 for ≤ pT3 vs pT4 and p = 0.153 for pT4 vs M1c in M–U). (**d**) IL5 was not significantly different according to peritoneal cancer burden (p = 0.017 in K–W; p = 0.024 for ≤ pT3 vs pT4 and p = 0.016 for pT4 vs M1c in M–U). Ordinates are plotted on a log scale. *K–W* Kruskal–Wallis test, *M–U* Mann–Whitney U test).

Of the ten cytokines examined, the levels of IL (interleukin)4, IL6, IL10, IL12 (IL12p70) and TGFB1 (transforming growth factor beta-1) increased with increasing peritoneal cancer burden (p < 0.005 by Kruskal–Wallis test) although the subgroup analyses mostly were not significant, whereas the levels of IL2, IL5, IL17A (IL17A homodimer), IFNG (interferon-gamma) and TNF (tumor necrosis factor) did not. In most cases, the levels of IL4 and IL12 were lower than the range covered by the reference standards (7.8 ~ 500 pg/mL), rendering the measurements meaningless; therefore, of the ten cytokines tested, IL6, IL10, and TGFB1 tended to increase with peritoneal cancer burden, and IL6 and IL10 were significantly different between the pT4 and M1c groups (Table 1; Fig. 2).

**Figure 3:**
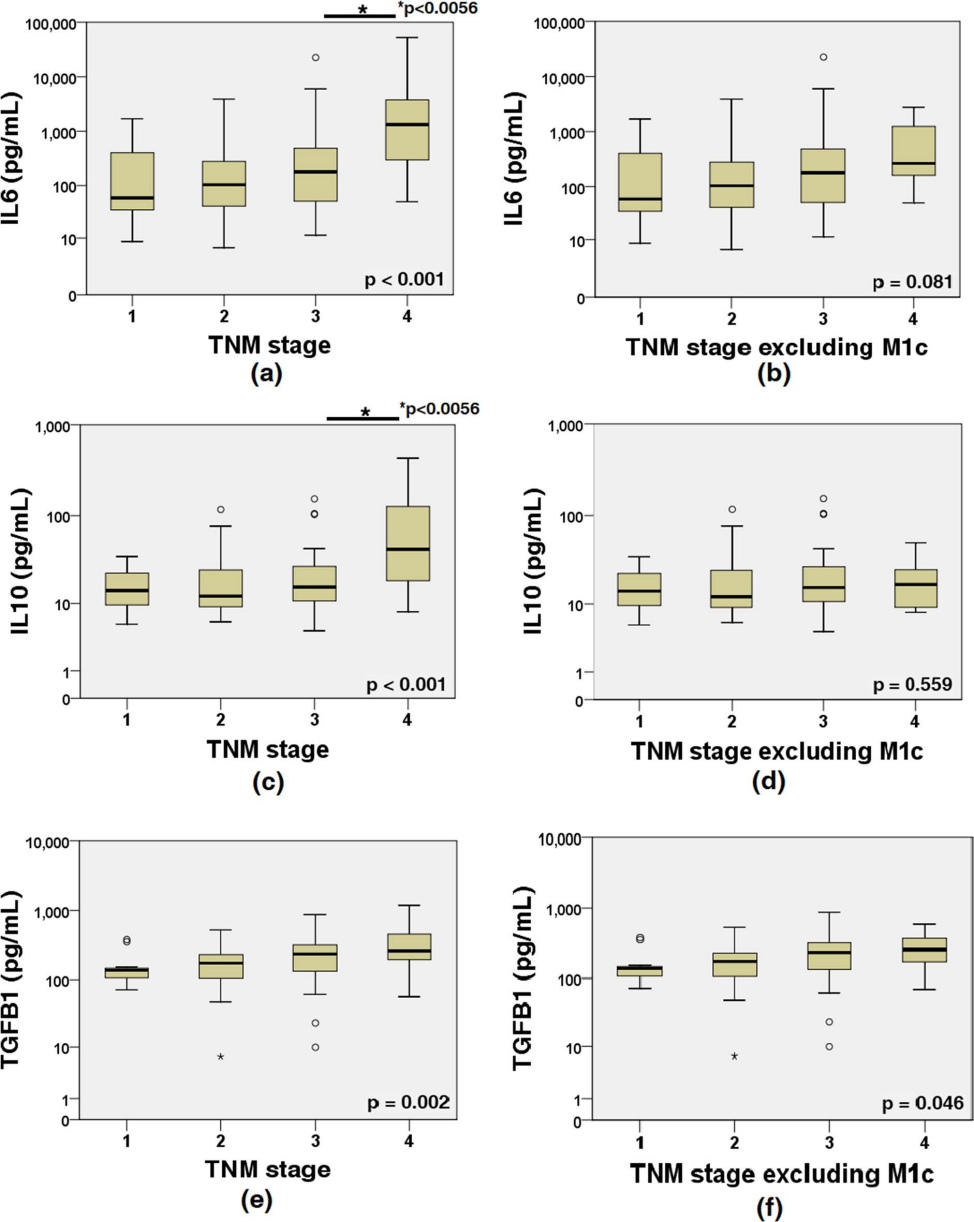
Cytokine concentrations in ascitic fluid according to TNM stage. (**a**) Peritoneal IL6 levels were higher in stage IV patients (p < 0.001 for stage III vs IV). (**b**) However, the peritoneal IL6 level of stage IV patients was not different if M1c patients were excluded (p = 0.170 for stage III vs IV). (**c**) The peritoneal IL10 level was higher in stage IV patients (p < 0.001 for stage III vs IV). (**d**) However, the peritoneal IL10 level of stage IV patients was not different if M1c patients were excluded (p = 0.860 for stage III vs IV). (**e**) Peritoneal transforming growth factor beta 1 (TGFB1) levels according to TNM stage (p = 0.130 for stage III vs IV). (**f**) TGFB1 levels according to TNM stage after excluding M1c patients (p = 0.532 for stage III vs IV). Ordinates are plotted on a log scale.

Although the levels of ascitic IL6 and IL10 between TNM stage III and IV patients were significantly different, they were not different when M1c group patients were excluded (Table 2; Fig. 3). These data indicate that concentrations of IL6 and IL10 in ascitic fluid are affected by peritoneal tumor burden regardless of nodal metastasis or hematogenous distant metastasis.

**Table 2:** Cytokine levels^¶^ in the ascites according to TNM stage.

	Stage I	Stage II	Stage III	Stage IV	*P*
**All patients (n = 143)**	**n = 12**	**n = 48**	**n = 43**	**n = 40**	
IL6 (pg/mL)*^,^^§^	59.0 (34.6, 658.3)	103.8 (40.9, 280.7)	179.0 (49.1, 490.0)	1,330 (288.8, 3834.0)	< 0.001
IL10 (pg/mL)*^,^^§^	14.2 (8.8, 23.2)	12.2 (8.6, 25.9)	15.6 (10.7, 29.6)	42.1 (17.9, 126.8)	< 0.001
TGFB1 (pg/mL)*	137.6 (91.5, 153.7)	174.8 (100.8, 232.3)	236.4 (133.4, 326.3)	262 (193.5, 477.9)	0.002
**Patients excluding M1c (n = 117)**	**n = 12**	**n = 48**	**n = 43**	**n = 14**	
IL6 (pg/mL)	59.0 (34.6, 658.3)	103.8 (40.9, 280.7)	179.0 (49.1, 490.0)	265.5 (156.5, 1291.3)	0.081
IL10 (pg/mL)	14.2 (8.8, 23.2)	12.2 (8.6, 25.9)	15.6 (10.7, 29.6)	16.9 (9.0, 25.8)	0.559
TGFB1 (pg/mL)	137.6 (91.5, 153.7)	174.8 (100.8, 232.3)	236.4 (133.4, 326.3)	258.2 (165.3, 392.3)	0.046

*IL* interleukin, *TGFB1* transforming growth factor beta1.

*p < 0.017, by Kruskal–Wallis test for stage I *vs.* stage II *vs.* stage III *vs.* stage IV.

^†^p < 0.0056, Bonferroni adjusted p-value by Mann–Whitney U test for stage I vs. stage II, two-tailed.

^‡^p < 0.0056, Bonferroni adjusted p-value by Mann–Whitney U test for stage II vs. stage III, two-tailed.

^§^p < 0.0056, Bonferroni adjusted p-value by Mann–Whitney U test for stage III vs. stage IV, two-tailed.

^¶^Median value (first quartile, third quartile).

### Patients with pT4 disease are at high risk of PR

PR by June 2019 in patients who underwent surgery with curative intent during the study period (August 1, 2009, to June 30, 2017) was surveyed based on data from medical records, regardless of ascites harvest (Table 3). There were 45 (5.8%) PRs and 112 (14.5%) systemic recurrences (SRs) among 775 patients undergoing curative surgery who had neither preoperative chemotherapy nor radiotherapy and who had neither retroperitoneal (Rb) rectal cancer nor M1c stage disease.

**Table 3:** Peritoneal and systemic recurrence rates^*^ during study period according to T stages in curatively resected patients.

	Number of patients during study period						Pt’s with ascites
T stage	T0	T1	T2	T3	T4	Total	T4
Recurrence^†^	(n = 33)	(n = 105)	(n = 89)	(n = 451)	(n = 97)	(n = 775)	(n = 62^‡^)
**PR**							
(−)	33	103	89	435	70	730	44
(+)	0	2	0	16	27	45	18
% PR(+)	0	1.9	0	3.5	27.8	5.8	29.0
**SR**							
(−)	33	99	87	372	72	663	42
*PR(−) in SR(−)*	*33*	*99*	*87*	*369*	*59*	*647*	*34*
*PR(*+*) in SR(−)*	*0*	*0*	*0*	*3*	*13*	*16*	*8*
*% PR(*+*) in SR(−)*	*0*	*0*	*0*	*0.8*	*18.1*	*2.4*	*19.0*
( +)	0	6	2	79	25	112	19
*PR(−) in SR(*+*)*	*0*	*4*	*2*	*66*	*11*	*83*	*9*
*PR(*+*) in SR(*+*)*	*0*	*2*	*0*	*13*	*14*	*29*	*10*
*% PR (*+*) in SR (*+*)*		*33.3*	*0*	*16.5*	*56.0*	*25.9*	*52.6*
% SR	0	5.71	2.25	17.5	25.8	14.5	31.1

*Recurrence of patients excluding palliative resection, preoperative chemo- or radiotherapy, retroperitoneal rectal cancer, M1c stage, operative mortality and follow up less than 1 month regardless of ascites harvest.

^†^PR: peritoneal recurrence; SR: systemic recurrence.

^‡^One patient with ascites but without IL10 data was included.

As expected, PR was most common (27 of 97, 27.8%) in pT4 group patients and was higher in patients with SR (14 of 25, 56%) than in those without SR (13 of 72, 18.1%) (*p* < 0.001, two-tailed χ^2^ test). PR also occurred in patients with T1 and T3 tumors; however, the frequency was very low, and most were accompanied by SR (100% in T1 (2 of 2) and 81.3% in T3 (13 of 16)). There was no difference in the PR rates between patients with pT4 with (29.0%, 18 of 62) and without (25.7%, 9 of 35) harvestable ascites (p = 0.726, two-tailed χ^2^ test). Therefore, patients with pT4 tumors were the most appropriate group in whom to investigate PR with respect to the mechanism and frequency.

### Ascitic IL10 is a prognostic marker of PR in pT4 group patients

**Figure 4:**
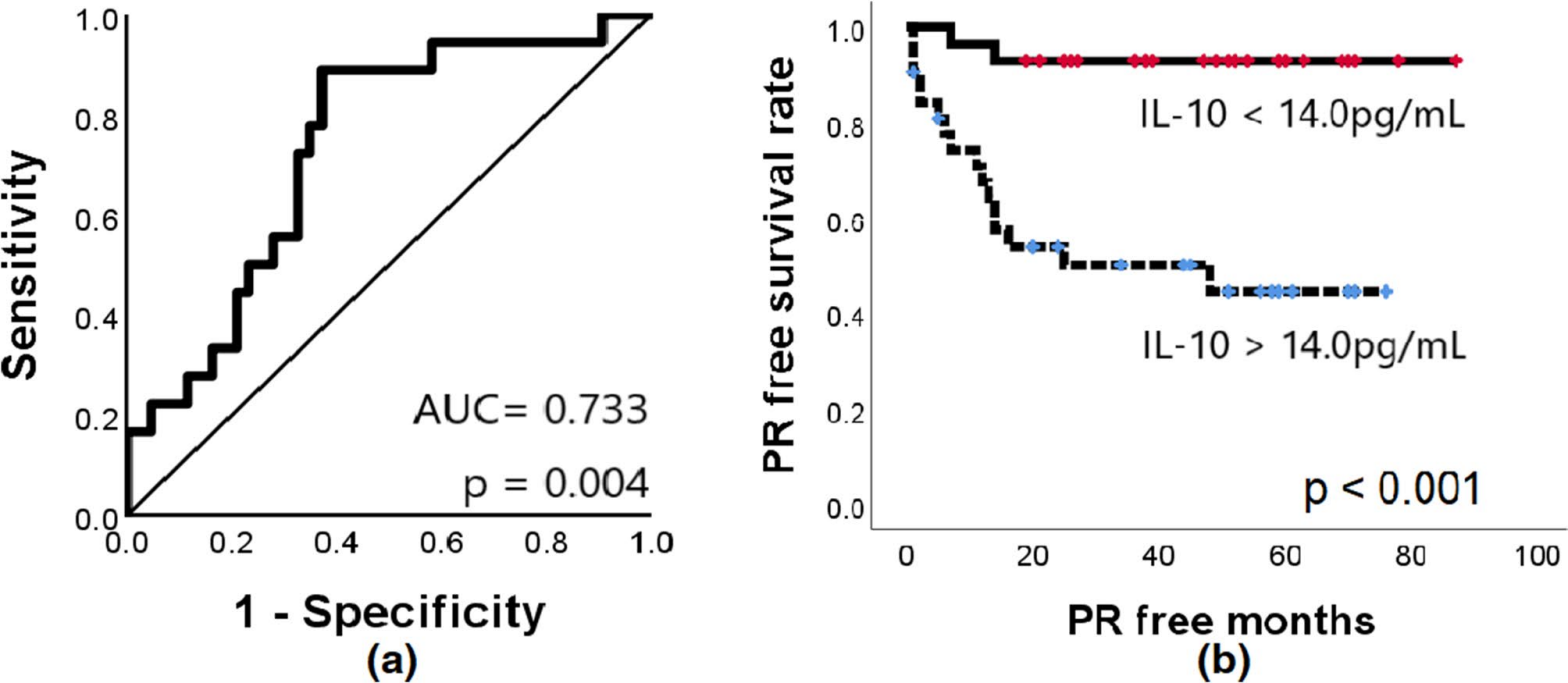
Ascitic IL10 level as a prognostic marker for peritoneal recurrence in patients with stage pT4 CRC. (**a**) Receiver operating characteristic curve for IL10 shows an AUC of 0.733 (p = 0.004). The relevant cut-off value is 14.0 pg/mL. (**b**) Peritoneal recurrence-free survival of patients with low (≤ 14.0 pg/mL) and high (> 14.0 pg/mL) peritoneal IL10 levels was significantly different (*p* < 0.001; log-rank test). *AUC* area under the curve, *PR* peritoneal recurrence.

Of the 79 patients in the pT4 group with available ascites (cohorts I and II; Fig. 1), one did not have the data on three cytokines (TGFB1, IL6 and IL10), 14 underwent palliative resection (without resection of distant metastasis), one was an operative mortality and two were lost to follow-up (less than 1 month). Of the remaining 61 patients who underwent curative resection, eighteen experienced PR at a median of 9 (range 1–48) postoperative months, with a median follow-up period of 39 (range 1–87) months. The median IL10 concentration in patients with pT4 tumors who experienced PR (27.8 pg/mL) was significantly higher than that in patients who did not (12.1 pg/mL; *p* = 0.004, Mann–Whitney U test). However, although the median IL6 concentration in pT4 group patients who experienced PR (294 pg/mL) was higher than that in pT4 group patients who did not (154 pg/mL), the difference was not significant (*p* = 0.066). Similarly, TGFB1 concentrations did not differ between patients with (292.7 pg/mL) and without (211.0 pg/mL) PR (*p* = 0.267, in cohort I only; determination of TGFB1 levels in cohort II was not possible due to technical problems). ROC curve analysis generated AUC (area under the curve) values of 0.733 for IL10 (*p* = 0.004), 0.651 for IL6 (*p* = 0.066), and 0.628 for TGFB1 (*p* = 0.267), and the calculated cut-off of IL10 concentration for predicting PR was 14 pg/mL (Fig. 4). The cumulative PR rates were 6.9% (2/29) for patients with low IL10 levels (≤ 14 pg/mL) and 45% (16/32) for those with high IL10 levels (> 14 pg/mL; *p* < 0.001, log-rank test). Furthermore, curves for PR according to time showed a significant difference between patients with high and low IL10 levels.

We next performed Cox regression analysis (backward Wald test), including other clinicopathological factors that are considered to be risk factors for recurrence, to examine whether increased IL10 was an independent risk factor for PR in pT4 patients (Table 4). Because IL6 and TGFB1 were highly correlated with IL10 (Pearson’s r = 0.640, p < 0.001 for IL6; Pearson’s r = 0.646, p < 0.001 for TGFB1), they were not included in the analysis. Univariable analysis identified gender, IL10 level, node metastasis and SR as significant risk factors for PR. In the multivariable analysis, including factors with *p* < 0.1 in univariable analysis, high IL10 level (hazard ratio (HR), 6.982; 95% confidence interval (CI) 1.541–31.628, *p* = 0.012) was a significant risk factor for PR, while gender (HR, 2.341; 95% CI 0.887–6.173, *p* = 0.086), node metastasis (HR, 0.582; 95% CI 0.203–1.668, *p* = 0.313) and SR (HR, 0.394; 95% CI, 0.152–1.022, *p* = 0.055) were not significantly associated with PR.

**Table 4:** Factors contributing to peritoneal recurrence in pT4 patients after curative resection (n = 61).

		Peritoneal recurrence (PR)				p			
		PR (+) (n = 18)		PR (−) (n = 43)		Univariable analysis			Multivariable analysis
Age (years, Mean ± SD)*		70.7 ± 12.6		67.0 ± 11.6		0.181			
**Gender**						0.007^‡^ 3.64 (1.42–9.30)			0.086
Male		8		32					
Female		10		11					
Body mass Index*						0.109			
Mean ± SD		20.5 ± 3.0		22.2 ± 3.7					
**IL10**^**†**^						0.002^‡^ 10.18(2.33–44.28)			0.012^§^
Low (≤ 14 pg/ml)		2		27					
High (> 14 pg/ml)		16		16					
**ASA score**						0.211			
ASA 1		3		9					
ASA 2		9		27					
ASA 3		6		7					
**Preoperative CEA (ng/mL)**						0.894			
≤ 5		10		20					
> 5		8		19					
**Operation method**						0.582			
Open surgery^¶^		9		19					
Laparoscopic surgery		9		24					
**Tumor location**						0.809			
Proximal (A-T)		9		21					
Distal (D-R)		9		22					
**Colon obstruction**						0.669			
(+)		5		9					
(−)		13		34					
Tumor size (cm)*						0.311			
Median (Q1, Q3)		7.0 (4.9, 9.5)		6.0 (5.0, 8.0)					
**Differentiation**						0.380			
WD/MD		13		35					
PD/UD/Muc		5		8					
**Venous invasion**						0.283			
Positive		5		8					
Negative		13		35					
**Perineural invasion**						0.397			
Positive		11		21					
Negative		7		22					
**Angiolymphatic invasion**						0.154			
Positive		13		25					
Negative		5		18					
**T4 stage**						0.219			
T4a		10		31					
T4b		8		12					
**Node metastasis**						0.047^‡^ 2.71(1.01–7.25)			0.310
Yes		12		18					
No		6		25					
TNM stage**						0.557			
II		6		21					
III		12		17					
IV		0		5					
**Chemotherapy**						0.252			
Yes		9		28					
No		9		15					
**Systemic recurrence**						0.013^‡^ 3.32(1.29–8.49)			0.055
Yes		10		9					
No		8		34					

*IL* interleukin, *A* ascending, *T* transverse, *D* descending,* R* rectum, *(Q1, Q3)* (first quartile, third quartile), *WD* well differentiated, *MD* moderately differentiated, *PD* poorly differentiated, *UD* undifferentiated, *Muc* mucinous.

*Age and BMI had normal distribution (p > 0.05, Shapiro–Wilk test) but tumor size did not. (p < 0.05 in PR(-)).

^†^The cut-off value obtained using the maximum value of Youden’s index (sensitivity = 0.889, specificity = 0.628).

^‡^p < 0.1, Univariable Cox regression.

^§^p < 0.05, Multivariable Cox regression.

^¶^Conversion cases from laparoscopic to open surgeries were included.

**Cox regression with Firth’s correction.

^††^Data from two patients were missed in BMI and four data were missed in CEA.

**Figure 5:**
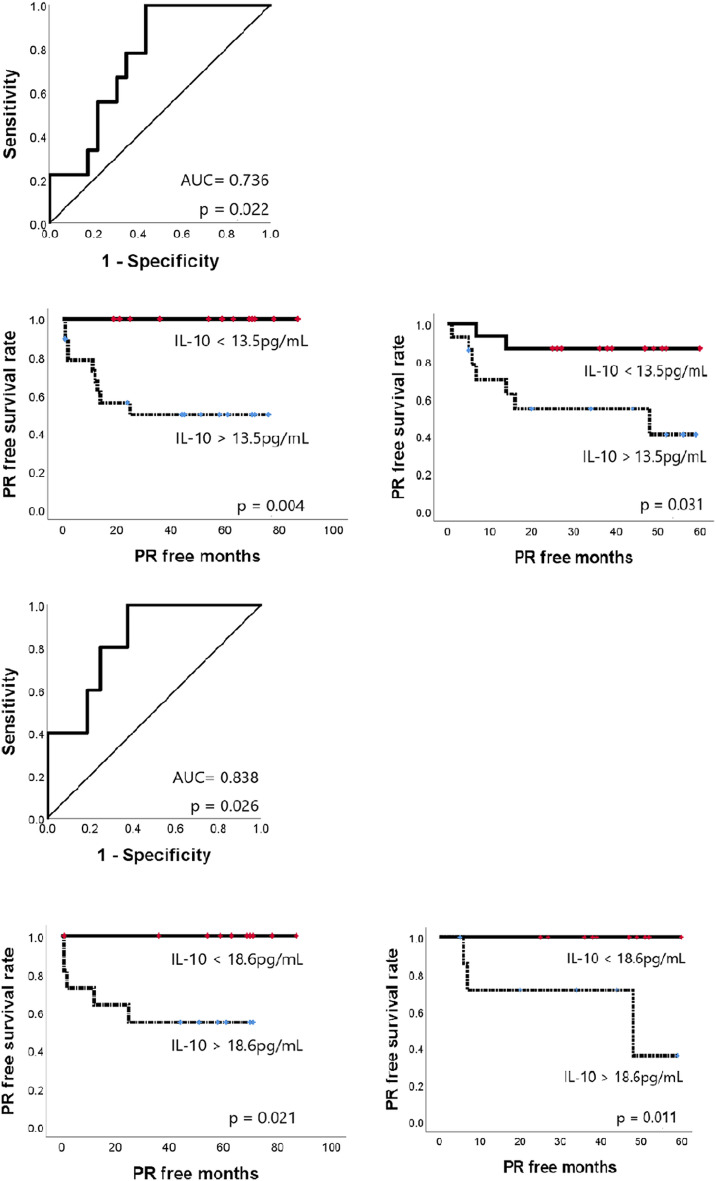
Classification performance of the IL10 cut-off model. (**a**) ROC curve from cohort I (n = 32) showed an AUC of 0.736 (p = 0.022). The relevant cut-off value was 13.5 pg/mL. (**b**) The difference in PR in cohort I was significant (p = 0.004; no PR of 13 for ≤ 13.5 pg/mL and 9 PR of 19 for > 13.5 pg/mL in cohort I). (**c**) The difference in PR in cohort II (n = 29) was also significant (p = 0.031; two PRs of 15 for ≤ 13.5 pg/mL and 7 PRs of 14 for > 13.5 pg/mL in cohort II). (**d**) The ROC curve from cohort I without SR (n = 21) showed an AUC of 0.838 (p = 0.026). The relevant cut-off value was 18.6 pg/mL. (**e**) The difference in PR in cohort I without SR was significant (p = 0.021; no PR of 10 for ≤ 18.6 pg/mL and 5 PR of 11 for > 18.6 pg/mL in cohort I). (**f**) The difference in PR in cohort II (n = 21) without SR was also significant (p = 0.011; no PR of 13 for ≤ 18.6 pg/mL and 3 PR of 8 for > 18.6 pg/mL in cohort II). *ROC* receiver operating characteristic, *AUC* area under the curve, *PR* peritoneal recurrence, *SR* systemic recurrence).

Although this was a relatively small study, we divided the data set of 61 patients into training (cohort I, n = 32) and test (cohort II, n = 29) groups to validate the classification performance of the IL10 cut-off model. The optimal cut-off value derived from cohort I was 13.5 pg/mL (AUC = 0.736, *p* = 0.022), which could distinguish between the low and high recurrence groups (p = 0.004) and divide cohort II into low and high recurrence groups (p = 0.031). Moreover, IL10 was also a significant factor for predicting PR, with a higher AUC value, when used for validation in patients with PR and without SR (Fig. 5).

## Discussion

The incidence of synchronous or metachronous PC is not well known; however, Santvoort et al.^13^ reported that 23% of T4 CRC patients had synchronous PC and 21% had metachronous PC, and Segelman et al.^14^ reported that 27.7% of T4 CRC patients had metachronous PC. In our series, there were 28 patients with synchronous PC of the 153 T4 patients (18.3%) who underwent resection of the primary lesion. Moreover, 27 of the 97 T4 patients (27.8%) who underwent curative resection were diagnosed with PR (Table 3). The overall incidences of synchronous PC and later PR were not very different from those previously reported. However, the risk factors of PR in patients of stage pT4 is poorly understood. Nagata et al. reported that poor differentiation, lymph node metastasis and preoperative CEA were independent risk factors for peritoneal recurrence in a larger cohort. We did not find such results, probably due to the small size of the patient cohort^15^.

If carcinoma cells escape immune surveillance by immunoediting, they can form a tumor; if not, they are destroyed by the immune system^12^. The mechanisms by which cancer cells escape immune surveillance include loss of antigenicity, loss of immunogenicity and suppression of antitumor immune responses^16^. Factors that suppress the immune response include IL10, TGFB1, indoleamine dioxygenase (IDO), soluble Fas ligand, and cellular components such as regulatory T-cells and myeloid-derived suppressor cells^6^. Activation of immune checkpoints can also suppress the immune response^17^. In this study, increased levels of IL10, IL6 and TGFB1 correlated with an increased tumor burden.

IL10 is produced not only by immune cells but also by cancer cells themselves^18^. Many studies have examined the ability of IL10 to suppress antitumor immunity. For example, IL10 secreted by peritoneal monocytes downregulates cytokine production and T-cell proliferation in ovarian cancers^19^. Patients with more advanced CRC have higher serum IL10 levels^20^, and serum IL10 has been shown to affect the prognosis of colon cancer patients^21^. In addition, Giacomelli et al.^22^ reported higher recurrence rates in patients with persistently high serum IL10 levels. However, those studies were based on measurements of IL10 in the serum, whereas our study is the first to measure IL10 levels in ascites, where peritoneal carcinoma cells grow, and to observe the prognosis of patients with PR. The IL10 levels presented herein are supported by other studies showing similar IL10 levels in ascites^23^. As an IL10 ELISA is far simpler and more convenient than detecting and quantitatively measuring free peritoneal cancer cells, so this may be a preferable method for assessing the risk of PR.

IL6, a multipotent proinflammatory cytokine, is known to be expressed in colon cancer tissues^24,25^ and plays a role in proliferation, metastasis and angiogenesis^26,27^. Because the immune response is a complex network of immune cells and molecules, IL10 and IL6 are only limited aspects of the immunosuppressive peritoneal environment. Detailed immunological mechanisms underlying peritoneal tumor growth, including tumor factors such as adhesion molecules^28^, should be examined through further investigations of this model.

The amount of peritoneal fluid varies from patient to patient but generally increases with increasing peritoneal tumor burden. Hydration before surgery made ascites sampling possible in most pT4 patients and in approximately half of ≤ pT3 patients unless pelvic adhesion or bleeding prohibited sampling. The minimum volume of ascites needed to measure the ten cytokines was 1.5–3 mL, and patients with ascites volumes less than that were excluded by secondary exclusion. Nevertheless, it is not evident whether IL10 is related to peritoneal tumor growth in patients without ascites.

There was doubt that the low IL10 levels in ≤ pT3 patients was due to a dilution effect in patients with less ascites. However, this is unlikely because hydration was performed in all surgical patients and some other cytokines did not show similar tendencies. Moreover, we could acquire sequential samples from three M1c patients who needed repeated aspirations to relieve abdominal distension. The IL10 concentrations were always higher in the later samples (more progressed PC) for all 3 patients. This finding supports that progression of peritoneal tumor burden is accompanied by increases in ascitic IL10 levels.

We evaluated the ascitic IL10 level at the time of laparotomy opening, not laparotomy closure, for postoperative peritoneal recurrence. Because ascites at the time of closing laparotomy is not only ascites but instead a mixture of ascites, blood and irrigation saline, it has little significance for the immune status of the peritoneal cavity. Therefore, the elevated peritoneal IL10 levels found in this study are thought to imply latent and microscopic peritoneal tumor implants containing tumor cells as well as immune cells, which could be accidentally eradicated within the removed surgical specimens or with postoperative chemotherapy and which would otherwise become peritoneal recurrence.

This study has some limitations. We examined only a small number of patients, and we did not include the assessment of peritoneal cancer cells themselves. In addition, we cannot explain the high IL10 levels in some T3 or lower patients. Finally, a practical cut-off value for IL10 and a standardized and effective way of acquiring ascites are needed. Despite these limitations, the present study is the first to measure the concentration of immune cytokines in ascites, the fluid that forms the microenvironment for progressing tumors in the peritoneal cavity.

This is a good human model for studying the immune response to colorectal tumor growth, in which ascites is ready for protein assay and cellular analysis. The assay of additional immune proteins (such as cytokines, chemokines, and growth factors), identification of the original cells of significant proteins, investigation of differences in detailed immune characteristics between pT4 patients with and without peritoneal recurrence and characterization of the spatial arrangement of each immune cell and cancer cell within peritoneal seeding nodules will provide much information on the immune response to cancer growth as well as immune suppression. Most of the patients in this study were microsatellite stable because we did not sort the patients according to MSI status. Therefore, we anticipate that further studies of this model will supply evidence of immunotherapy for microsatellite-stable colorectal cancers, which is not indicated with the current immunotherapeutic, anti-PD-1.

## Conclusion

Peritoneal IL10 concentration correlates with peritoneal tumor burden in patients with CRC. Ascitic IL10 is a prognostic marker of PR in patients with stage T4 CRC following curative-intent resection. More immune factors, including immune cell functions, should be explored in this model with a larger cohort to better understand the immunological characteristics that affect intraperitoneal CRC growth.

## Materials and methods

Ascites samples were collected prospectively from patients with CRC (adenocarcinoma) who underwent surgery at the Seoul National University Boramae Medical Center since August 2009. Patients undergoing surgery from August 1, 2009, to June 30, 2017, were enrolled and surveyed for recurrence until June 30, 2019 (Fig. 1).

This study was approved by the Ethics Committee of Boramae Medical Center (IRB No. 06-2009-63) and performed in line with the principles of the Declaration of Helsinki. Informed consent was obtained from all individual participants included in the study.

### Ascites collection

To facilitate ascites sampling, the patients were supplemented with intravenous fluid the day before surgery to avoid dehydration during the fasting or bowel preparation stages. After general anesthesia, the operating table was tilted into the reverse Trendelenburg position to allow the ascites to run into the Douglas pouch. Care was taken to ensure that blood or tissue fluid from the incision site did not flow into the peritoneal cavity during laparotomy incision or laparoscopic port insertion. As soon as the peritoneal cavity was opened, ascites samples were aspirated from the Douglas pouch and transferred to polypropylene tubes. Fibrin materials and cellular debris were removed by centrifugation, and ascites was transferred to Eppendorf tubes, which were frozen at − 80 °C. Only ascites (not peritoneal irrigation fluid) was used.

Patients whose tumors were located below the peritoneal reflection (Rb rectal cancer), those who had undergone preoperative chemotherapy or radiotherapy, and those in whom the ascitic cytokines could have been affected by inflammation other than that caused by the cancer itself (such as intestinal perforations, peritumoral abscesses, fever, or leukocytosis) were excluded (primary exclusion). All the other patients were candidates for ascites sampling. However, some patients had pelvic adhesions prohibiting ascites collection, others had insufficient amounts of ascites fluid, and others presented bleeding during ascites collection, which can affect the concentrations of ascitic cytokines. These patients were also excluded from ascites collection (secondary exclusion) (Fig. 1).

Additionally, we collected ascites from M1c patients who were not surgical candidates but required aspiration of malignant ascites to reduce abdominal distension to include a sufficient number of patients with macroscopic peritoneal tumors.

### Cytokine assays

We selected ten cytokines that were frequently evaluated in immune responses. The levels of IL2 (555190, BD Biosciences, San Jose, CA), IL4 (88–7046, eBioscience, San Diego, CA), IL5 (555202, BD Biosciences), IL6 (555220, BD Biosciences), IL10 (555157, BD Biosciences), IL12p70 (88-7126, eBioscience), IL17A homodimer (88-7176, eBioscience), TNF (555212, BD Biosciences), IFNG (555142, BD Biosciences), and TGFB1 (acid activated, 88-8350, eBioscience) were measured using commercially available enzyme-linked immunosorbent assay (ELISA) kits, according to the manufacturer’s instructions. The cytokine assays for cryopreserved ascites were performed in several batches, as appropriate numbers of samples for one ELISA plate (10 ~ 30 samples) were collected. The reliability of ELISA for ascites was assessed retrospectively using the coefficient of variation and intraclass correlation coefficient for the duplicated wells as well as repeated measurements of the samples. Detailed procedures and assessments of the reliability of ELISA are described in the supplementary method file (Supplementary Methods).

### Patient grouping for the assessment of changes in cytokines

The patients were classified into three groups according to the extent of tumor exposure and growth in the peritoneal cavity (based on pathological results) as follows: no tumor cells (pT3 or lower T stages), microscopic tumor cells (pT4), and gross tumors (M1c). In the ≤ pT3 group patients, the primary carcinoma had not penetrated the serosa and there was no peritoneal seeding. In the pT4 group patients, carcinomas were exposed through the serosa of the colon without peritoneal seeding. In the M1c group, there were patients with a few localized peritoneal seeding nodules around the primary lesion or with multiple peritoneal seeding nodules throughout the peritoneum. Peritoneal metastatic carcinoma lesions in the M1c group were confirmed by pathological examination during the operation. Pathological stages were classified according to the 8th edition of the AJCC cancer staging manual. We reviewed pathologic slides of some patients from an earlier period of the study to clarify N1c and T4ab.

From August 2009 to January 2014 (cohort I), we collected ascites from 206, 39, and 26 (19 surgical and 7 nonsurgical) patients in the ≤ pT3, pT4, and M1c groups, respectively. Since February 2014, we also excluded patients with clinical T1 (cT1) or T2 (cT2) from ascites harvest because harvesting too much ascites in the ≤ pT3 group was not necessary (Fig. 1).

### Patient follow-up and recurrence

The patients were treated and followed up regularly after surgery. Postoperative chemotherapy was recommended and performed when indicated according to the NCCN (National Comprehensive Cancer Network) guidelines. However, some patients rejected chemotherapy. If the patient had even one cycle of scheduled chemotherapy, he or she was considered to have received chemotherapy. Serum carcinoembryonic antigen (CEA) was checked, and an abdominal computed tomography (CT) scan was conducted three or four times per year for patients with ≥ TNM stage II for the first 2 years; this was repeated twice a year for the next 3 years if there was no evidence of recurrence. PR was determined as follows: by surgical biopsy; when at least two serial images (CT or positron emission tomography scan) indicated the growth of a mass suggestive of PR; when a peritoneal mass appeared in a patient with elevated serum CEA levels but without accompanying distant metastasis; or when the size and number of recurrent masses were reduced by chemotherapy. Time to PR was defined as the time of the first recognition of a mass in imaging studies, which was determined to be PR. SR was determined similarly using CEA, imaging modalities, and surgical biopsy.

### Statistical analysis

To examine the normality assumption for continuous variables (cytokines), the Shapiro–Wilk test was performed. The cytokine levels among the groups were compared using the Kruskal–Wallis test, and the Mann–Whitney *U* test was used for post-hoc analysis. The risks of PR with T stages were compared using the χ^2^ test. To examine the ability of IL10 to predict PR, receiver operating characteristic (ROC) curve analysis was performed, and the cut-off value for IL10 was determined based on the maximum value of the Youden Index (J = sensitivity + specificity – 1).

Peritoneal disease-free survival was calculated using the Kaplan–Meier method, and the groups were compared using the log-rank test. To assess which factors were associated with PR, univariable and multivariable Cox regression models were applied, and we used Firth’s bias-correcting penalized maximum likelihood method^29^ for TNM stage due to the small sample size. Factors considered in the multivariable Cox regression model were selected from the univariable model (p < 0.1). In addition, to assess the proportional hazards assumption, Grambsch and Therneau’s test based on Schoenfeld residuals was used^30^. All statistical analyses were performed using SPSS version 20 (IBM Inc., Somers, NY, USA) and SAS software, version 9.4 (SAS Institute, Cary, NC, USA), with *p* < 0.05 considered significant. For multiple comparisons, *p* values were adjusted using Bonferroni correction using significance values derived by dividing the *p* value by the number of tests.

## Supplementary Information

Supplementary Information

## Data Availability

All data generated or analyzed during this study are included in this published article as Supplementary Information files.
