# Supplementary material for: Interleukin 10 level in the peritoneal cavity is a prognostic marker for peritoneal recurrence of T4 colorectal cancer
Source: Sci Rep. 2021 Apr 28;11:9212. doi: 10.1038/s41598-021-88653-2 (PMC8080840; doi:10.1038/s41598-021-88653-2)
Supplement: Supplementary file 1 — Supplementary Information [file 41598_2021_88653_MOESM1_ESM.docx]

**<Supplementary Methods>**

**Interleukin 10 level in the peritoneal cavity is a prognostic marker for peritoneal recurrence of T4 colorectal cancer**

Seung-Yong Jeong, MD, PhD^1^, Byeong Geon Jeon, MD, PhD ^2^, Ji-Eun Kim, MSc^3^,

Rumi Shin, MD, PhD^3^, Hye Seong Ahn, MD, PhD^3^, Heejin Jin, BSc^4^,

Seung Chul Heo*, MD, PhD^3^

**1. ELISA Solutions**

(In case of eBioscience ELISA kits, each buffers are supply within the kits)

1) Coating Buffer - 0.1 M Sodium Carbonate, pH 9.5

2) Assay Diluent- PBS with 10% Fetal Bovine Serum, pH 7.0.

3) Wash Buffer - PBS* with 0.05% Tween-20.

4) Substrate Solution - Tetramethylbenzidine (TMB) and Hydrogen Peroxide.

5) Stop Solution - 2 N H2SO4

**2. ELISA Procedures (Sandwich ELISA)**

The precise procedure may be different according to the kits. This is the summarized procedures from the manufacturer’s manuals from BD Bioscience. (<https://www.bdbiosciences.com/us/applications/research/t-cell-immunology/th-2-cells/immunoassays/elisa/human/human-il-10-elisa-set/p/555157>) and eBioscence (<https://assets.thermofisher.com/TFS-Assets/LSG/manuals/MAN0017439_88-7176_HuIL-17A-ELISA_PI.pdf> )

1) Coat 96-microwells with 100 µL per well of Capture Antibody diluted in Coating Buffer. For recommended antibody coating dilution, see lot-specific Instruction/Analysis Certificate. Seal plate and incubate overnight at 4° C.

2) Aspirate wells and wash 3 times with 300 µL/well with Wash Buffer. After the last wash, invert plate and blot on absorbent paper to remove any residual buffer.

3) Block plates with 200 µL/well Assay Diluent. Incubate at room temperature for 1 hour.

4) Aspirate/wash as in step 2.

5) Prepare standard and sample.

a. Prepare a top standard from the stock standard.

b. Add 300 µL Assay Diluent to 6~7 empty tubes according to the manufacturer’s guide.

c. Perform serial dilutions by adding 300 µL of each standard to the next tube and vortexing between each transfer.

d. Sample preparation for TGF-beta assay (acid activation): Add 20 μL of 1N HCl per 100 μL of each sample and incubate 10 minutes at room temperature, then neutralize with 20 μL of 1N NaOH.

6) Pipette 100 µL of each standard, sample, and control into appropriate wells. The samples and standards are always duplicated (put a sample in two wells). Seal plate and incubate for 2 hours at RT.

7) Aspirate/ wash as in step 2, but with 5 total washes.

8) Add 100 µL of Working Detector (Detection Antibody + Streptavidin-HRP reagent) to each well. Seal plate and incubate for 1 hour at RT. (Or add detection antibody and incubate sealed plate for 1 hour at room temperature. Then, after washing 5 times, add Avidin-HRP and incubate 30 minutes for eBioscience kits.)

9) Aspirate/ wash as in step 2, but with 7 total washes.

10) Add 100 µL of Substrate Solution to each well. Incubate plate (without plate sealer) for 15~30 minutes (according to the manufacturer’s guide) at room temperature in the dark.

11) Add 50 µL of Stop Solution to each well.

12) Read absorbance (OD: Optical density) at 450 nm within 30 minutes of stopping reaction.

13) Take the average of OD’s in the duplicated wells. Build a linear equation of the known concentrations of standards versus Log Mean OD using the applications in Microsoft Excel. Calculate the concentrations of the samples from the mean OD of each samples using the linear equation. (Figure 1) For TGF-beta, the measured concentration should be multiplied by 1.4 due to dilution during the acid activation procedures.


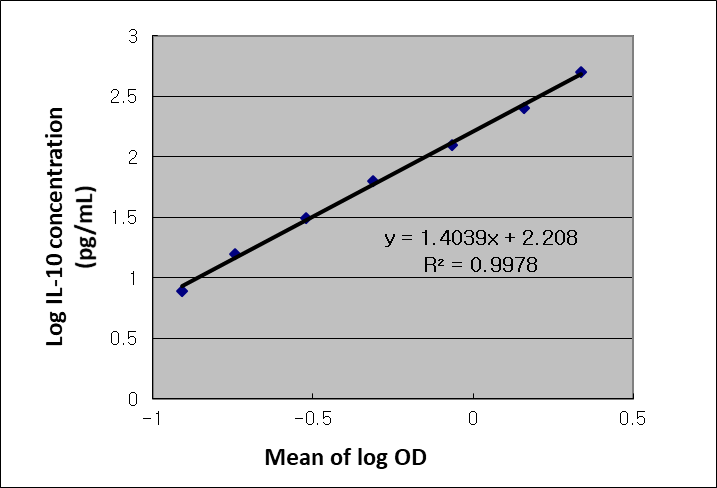


<Figure 1>

**3. Quality Maintenance**

1) The standards of each ELISA kit were prepared and stored according to the manufacture’s manual.

2) Ascites samples were centrifuged (for 5 minutes at 1,500rpm) after harvest to remove fibrin materials and cellular components, then placed 1 mL or less amount in each Eppendorf tube and stored in -80°C. The thawing and freezing were allowed maximum of 3 times for each Eppendorf tube to minimize protein destruction.

3) Frozen sample were thawed at room temperature just before the sample loading on ELISA plate. Eppendorf tubes were always vortexed prior to sample loading. After loading, the tubes were immediately re-frozen.

4) If a measured absorbance is higher than that of the highest standard concentration, that measurement was performed again with dilution of ascites samples using 1x PBS.

5) We did not perform quality assessment before the ELISA assay because we utilized commercially available assay kits. However, we retrospectively assessed the quality of ELISA assay by comparing the two absorbances (OD) from the duplicated wells at an assay as well as comparing the concentrations of same samples from repeated ELISA measurement using the distribution of coefficient of variation (CV = Standard deviation/Mean) and Intraclass correlation coefficient (ICC).

i) The mean of the CV’s from the two ODs of duplicated wells in 1,301 measurements (first measurement of 10 cytokines in 172 patients) in this research was 3.58% and 93.4% (1,215 of 1,301) was less than 10%. (Figure 2) The ICC of the paired ODs had high correlation in this


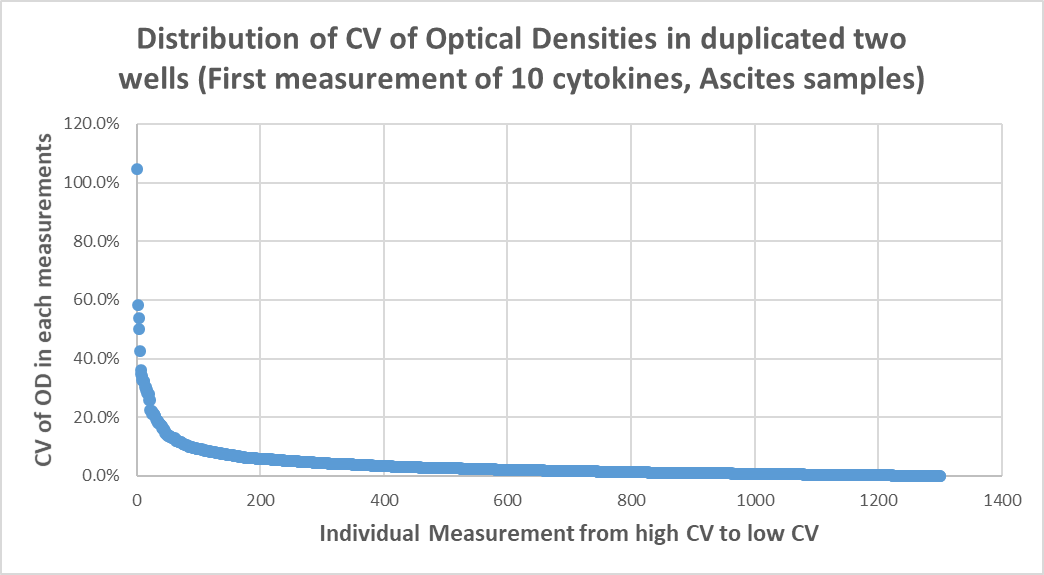


<Figrue 2>

measurement. (ICC(2,1) = 0.998 (95% CI = 0.998 – 0.998), p < 0.001) (Figure 3)


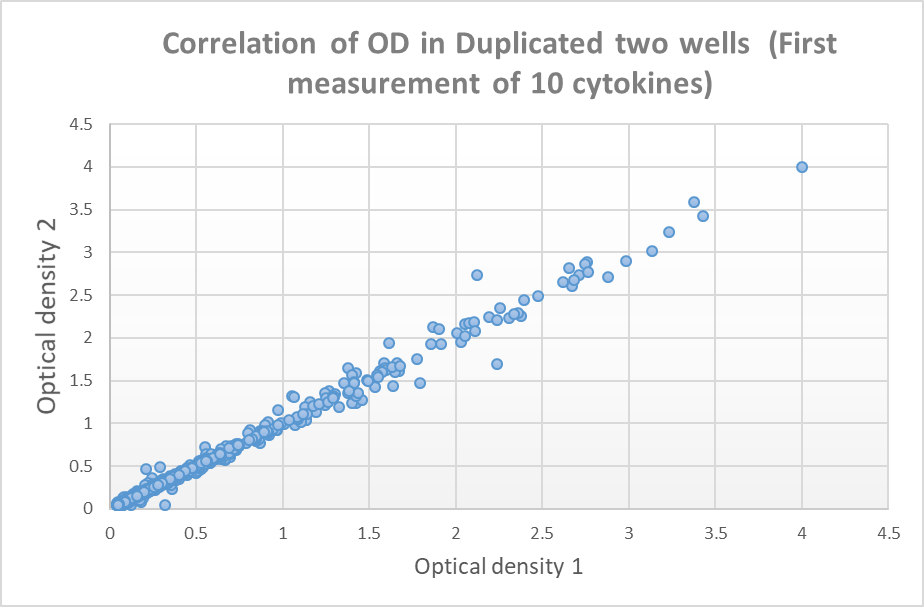


<Figure 3>

The distribution of CV of paired OD from the duplicated wells of first measurement of IL10 in this experiment is shown in Figure 4. The mean of the CV’s from ODs from the duplicated wells of 171 measurements is 3.8%, and 92.4% (158 of 171) of measurements had CV’s less than 10%.


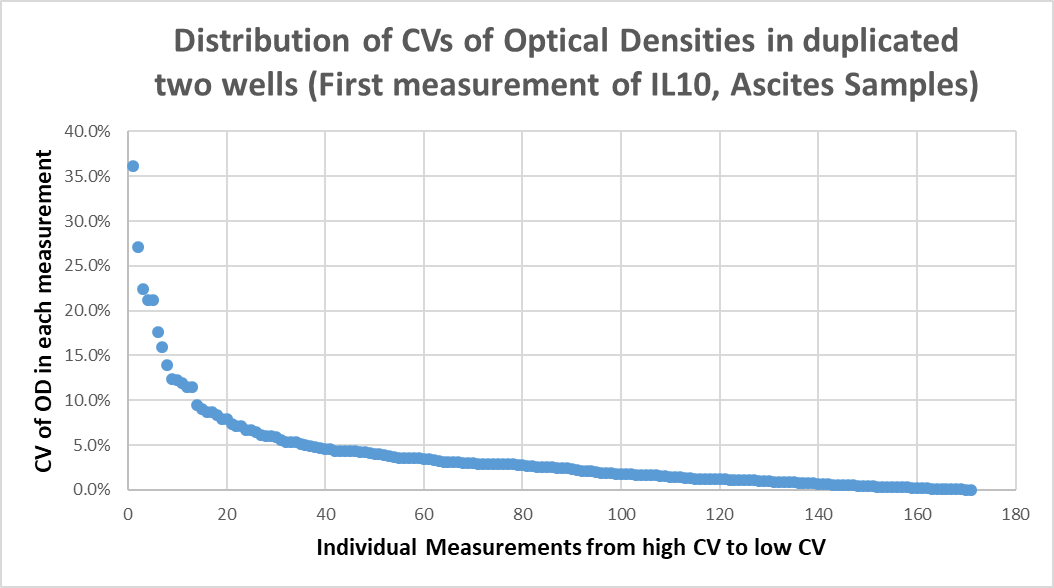


<Figure 4>

The ICC of the paired ODs from 171 IL10 measurement was

ICC(2,1) = 0.998 (95% CI = 0.997 – 0.998), p < 0.001 (Figure 5)


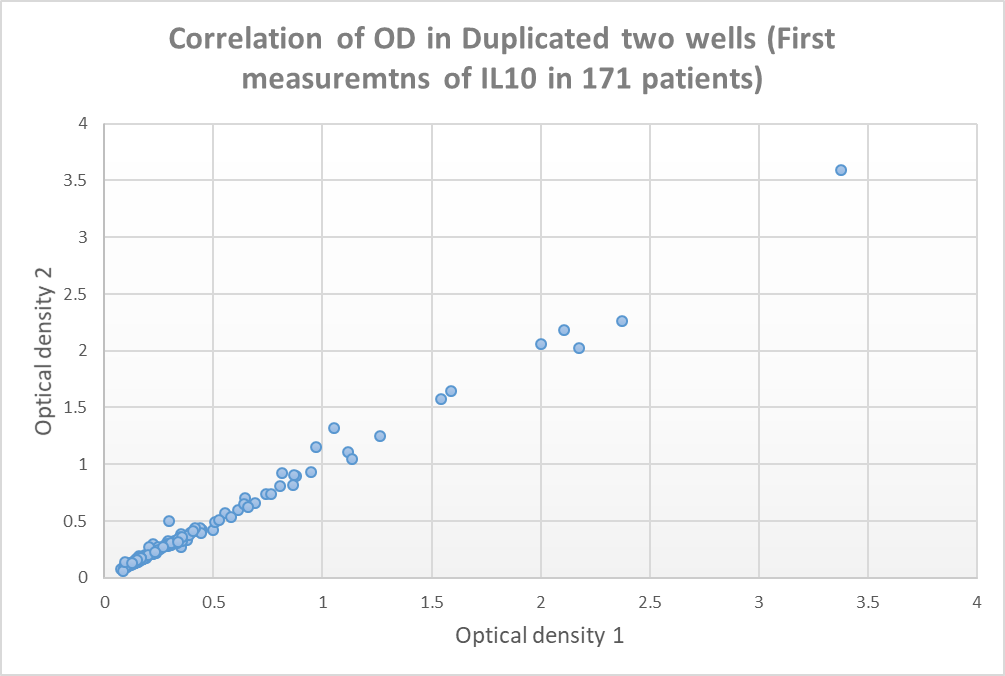


<Figure 5>

ii) We had repeated ELISA assay in some samples due to; re-measurement of the concentrations in samples with high CV from the two ODs of duplicated wells of previous measurement, to test the differences of repeated thawing and freezing, to test the long-term freezing effects and to test the reproducibility in regular samples. But that was not performed systematically due to the shortage of human samples. The reproducibility of IL10 concentration in the first and second measurement was assessed by CV and ICC. The mean of the CV’s of the two concentrations in 104 samples was 15.5% and 47.1% (49 of 104) of samples had CV less than 10% and 28.8% of samples (30 of 104) had CV of 10~20%. (Figure 6) The ICC of those samples was;

ICC (2,1) = 0.927 (95% CI = 0.893 – 0.951), p <0.001 (Figure 7)

Considering that this is mostly resulted from samples of deviation or of repeated thawing and freezing, that is acceptable. For the samples of large discrepancies, we repeated the ELISA (up to five times if ascites samples were available) and final IL10 concentration was determines by the average of them.


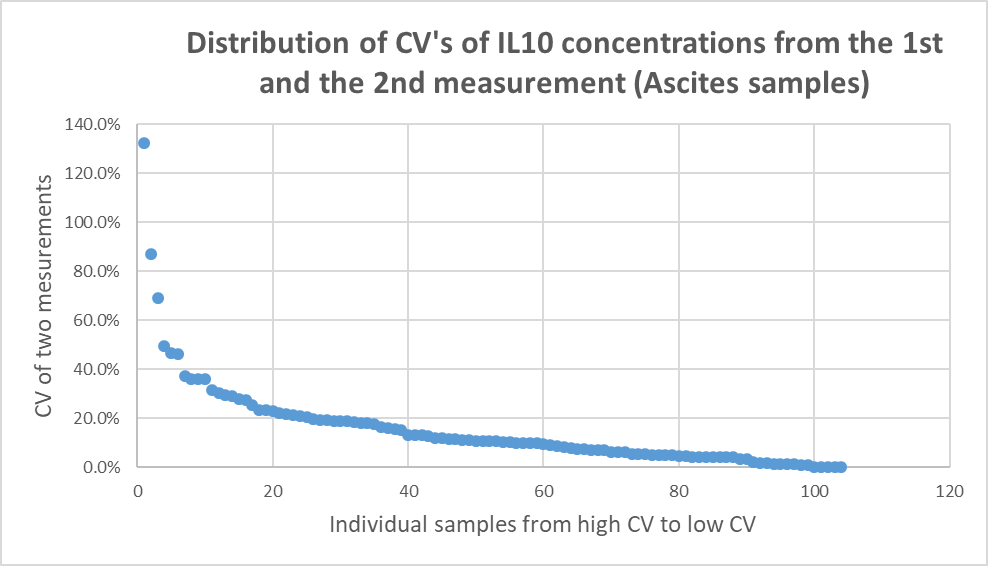


<Figure 6>


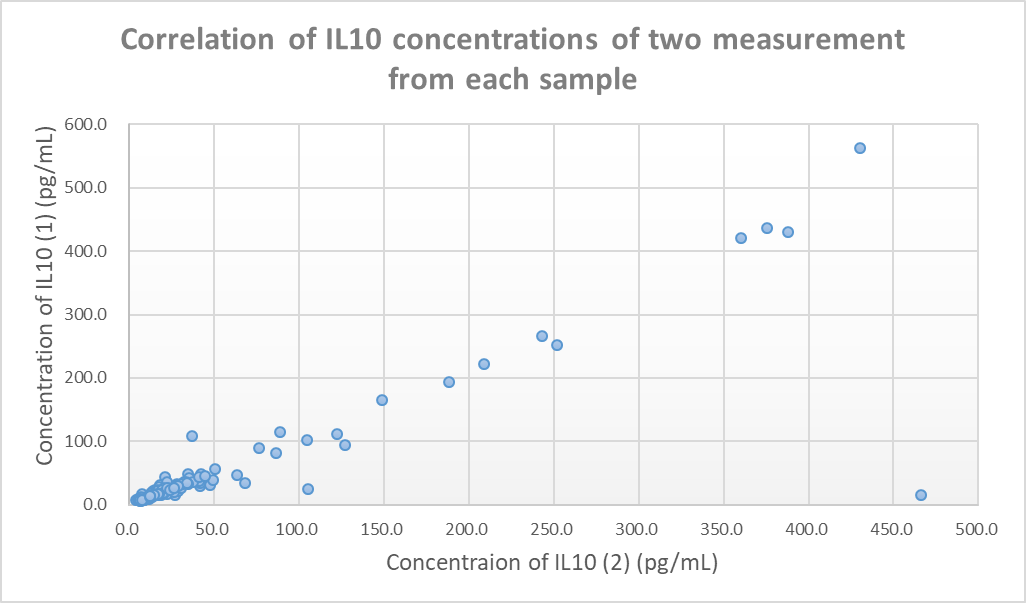


<Figure 7>

6) All the ELISA assays were performed by Kim J-E and assessment of peritoneal recurrences were performed by Shin R and Ahn HS without information of cytokine results.
